# Mediating Effect of Self-Cognitive Oral Health Status on the Effect of Obstructive Sleep Apnea Risk Factors on Quality of Life (HINT-8) in Middle-Aged Korean Women: The Korea National Health and Nutrition Examination Survey

**DOI:** 10.3390/life12101569

**Published:** 2022-10-09

**Authors:** Yu-Rin Kim

**Affiliations:** Department of Dental Hygiene, Silla University, Busan 46958, Korea; dbfls1712@silla.ac.kr; Tel.: +82-10-6686-8130

**Keywords:** oral health, obstructive sleep apnea, quality of life, women

## Abstract

Background: Obstructive sleep apnea and oral health are highly correlated with quality of life. The purpose of this study is to determine the mediating effect of self-cognitive oral health status on the effect of obstructive sleep apnea (OSA) risk factors on quality of life in middle-aged Korean women using the 2019 National Health and Nutrition Examination Survey data. Methods: A hierarchical regression analysis was performed on the mediating effect of self-cognitive oral health status on the effects of OSA risk factors on health-related quality of life (Korean health-related quality of life instrument with 8 items; HINT-8). Results: Self-cognitive oral health status as a parameter had a significant effect on quality of life (β = 0.713, *p* < 0.001). Compared to the effect of the OSA risk factor of the second stage on the quality of life (β = −1.329, *p* < 0.001), the input of the third stage of self-cognitive oral health was partially mediated (β = −1.280, *p* <0.001). Conclusions: Therefore, if middle-aged women have OSA risk factors, they should try to improve their quality of life by managing oral health as well as OSA treatment.

## 1. Introduction

The average human life span has been increasing recently, and interest in quality of sleep related to quality of life is consequently increasing as well. There are two types of indicators of sleep: quantitative indicators such as sleeping hours and number of awakenings during sleep and qualitative indicators such as degree of rest and satisfaction with sleep. Of these two aspects, quality of sleep is closely associated with health, life satisfaction and emotional states such as anger and depression [1]. This sleep disorder is one of the most common symptoms presented by 25–50% of women in menopause, which is a very high percentage compared to about 15% of the general population [2,3]. The high incidence of sleep disorders in menopausal women has been attributed to hormonal changes, while symptoms such as hot flashes (postmenopausal flushing), anxiety, and fatigue lead to sleep disorders [4]. These symptoms eventually reduce quality of life for middle-aged women. Thus, it is very important to improve sleep quality in order to achieve better quality of life [5]. 

Obstructive sleep apnea (OSA), a typical sleep disorder, is known to be over twice as common in men than in women, but its prevalence among postmenopausal women is similar to that of men [6]. OSA is a relatively common disease wherein breathing becomes irregular due to repeated narrowing or obstruction of the airways during sleep [7]. OSA patients also experience problems such as snoring, frequent wakefulness, and nocturia during sleep. Fatigue and cognitive impairment also appear due to lack of sleep, which increases the risk of various accidents. Left untreated, OSA increases the risk of developing cardiovascular disease [7]. One of the factors considered to cause increased postmenopausal OSA in middle-aged women is weight increase [8]. Neck circumference and weight gain can contribute to sleep apnea after menopause. In recent studies, the correlation between sleeping hours and weight gain is emphasized [9]. As sleep efficiency is reduced, lack of sleep induces appetite, which leads to higher incidence of respiratory problems during sleep along with weight gain. The decrease in estrogen and progesterone after menopause may also play a role. It is known that female hormones protect against breathing problems related to sleep disorder, while male hormones promote related diseases [10]. As such, although the risk factors for OSA among middle-aged women after menopause increase, there are very few cases where they are recognized as a disease and subsequently treated. Therefore, early detection and treatment of OSA is vital. One of the treatments for OSA is continuous positive airway pressure (CPAP), but this treatment’s effect is low due to low patient compliance [11]. Uvulopalatopharyngoplasty performed in otolaryngology is a procedure that expands the upper airway, and the success rate of long-term observation is only about half [12]. 

For OSA patients with difficulty controlling behavior, or when CPAP or surgical treatment cannot be performed or the patient refuses the treatment, the American Sleep Disorders Association recommends wearing an oral appliance [13]. Treatment of OSA is carried out in various medical departments including dentistry, because the causes of OSA are complex. It is known that the representative anatomical causes of OSA are mandibular retraction (retrognathia), narrow and deep palate, and long soft palate [14,15]. However, since it has been reported that one or more non-anatomical mechanisms are contributing to the pathogenesis of OSA in about 70% of OSA patients [14], it is necessary to check the oral condition a person is aware of. 

Ultimately, OSA and oral health are highly correlated with quality of life. Since the prevalence of OSA is significantly lower in female patients than in male patients [16], studies related to OSA to date have mostly been conducted on male patients or involving both men and women. Studies with only female patients are very few. Moreover, there are no large-scale studies conducted using data from the Korea National Health and Nutrition Examination Survey (KNHANES). OSA risk factors including potential OSA patients as well as patients diagnosed with OSA were likewise used as variables in this study.

Therefore, this study aims to confirm the general health status and quality of life characteristics of middle-aged Korean women according to OSA risk factors. It also seeks to determine the mediating effect of self-cognitive oral health status on the effects of OSA risk factors on quality of life. We intend to use the findings as basic data for programs to promote the importance of sleep and oral health in improving the quality of life of middle-aged Korean women.

## 2. Materials and Methods

### 2.1. Participants and Data Collection

**Figure 1:**
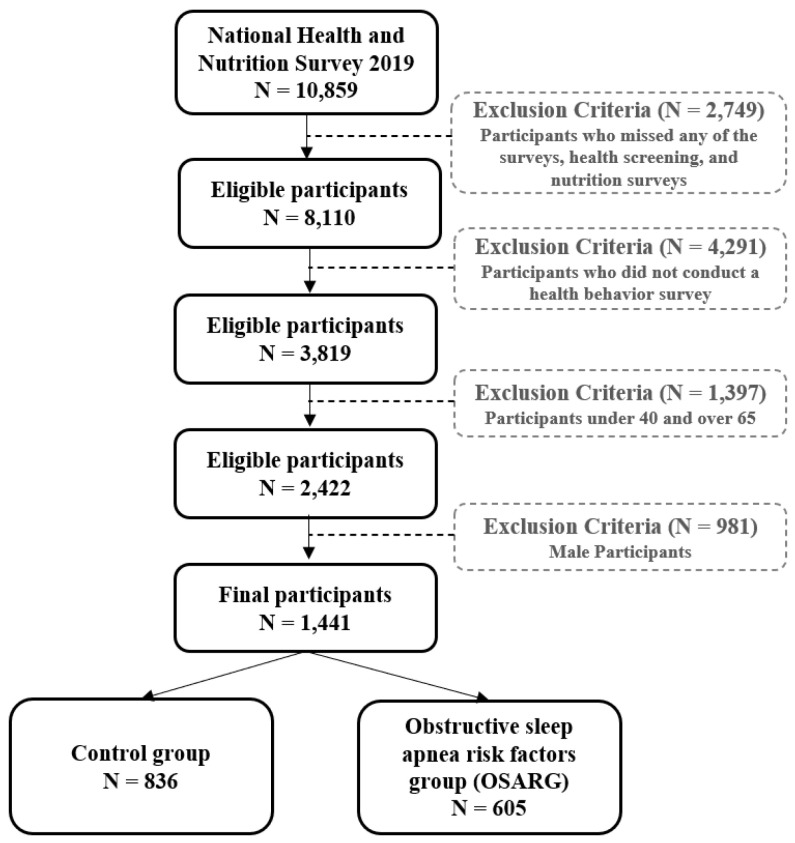
Flow of study population.

This study used data from the 2019 Korea National Health and Nutrition Examination Survey. Using a two-step stratified colony sampling method, the sampling frame was stratified and the living space ratio, the age of the householder, the ratio of single-person households, etc. were adopted as an intrinsic stratification criterion. In the 2019 survey, a total of 10,859 and 8110 people participated in at least one health survey, examination, and nutrition survey. Of the 3819 people in the health behavior investigation (self-administered survey) excluding missing values, the final 1441 people with female subjects only from the 2422 people aged 40 to 64 years old were analyzed (Figure 1). This study was approved by the Institutional Review Board (IRB; 2018-01-03-C-A) in consideration of the collection of human-derived materials and the provision of raw data to a third party.

### 2.2. Tools

#### 2.2.1. Covariance

##### Sociodemographic Characteristics

Age, marriage, education, income, economic activity status, drinking, and smoking were confirmed through the Korea National Health and Nutrition Examination Survey. Age was divided into ‘40–49 years old’, ‘50–59 years old’, ‘60–64 years old’; marriage was ‘married’ and ‘single’; and education was ‘below elementary school graduate’, ‘middle school graduate’, ‘high school graduate’, and ‘university graduate or higher’. Income was divided into quantiles: ‘low’, ‘low-middle’, ‘middle-high’, and ‘high’. Economic activity was divided into ‘employed’ and ‘unemployed’. With regard to drinking, the female binge drinking frequency variable was used, with 1 being ‘not drinking’, 2 being ‘less than once a month’, 3 being ‘about once a month’, 4 being ‘about once a week’, and 5 being ‘almost every day’. Higher scores indicated higher frequency of drinking. For smoking, the current smoking rate was confirmed, and 0 was classified as ‘past smoking or non-smoking’ and 1 as ‘current smoking’.

##### Systemic Health Status

Systemic diseases that affect OSA include hypertension, dyslipidemia, diabetes, cervical cancer, depression, allergic coryza, sinusitis, and tympanitis. The diseases were confirmed via a doctor’s diagnosis, and body mass index, neck circumference, and age at menopause were also confirmed.

#### 2.2.2. Self-Cognitive Oral Health Status

Self-cognitive oral health status was surveyed using a 5-point scale, with 1 being ‘very good’, 2 being ‘good’, 3 being ‘average’, 4 being ‘bad’, and 5 being ‘very bad’. A higher score means worse health. In this study, reverse coding was performed for readability, and a higher score indicated that the oral health status perceived by the person is very good.

#### 2.2.3. HINT-8

The Korea Disease Control and Prevention Agency (KDCA) developed HINT-8 as a tool to more accurately measure Koreans’ health-related quality of life [17]. HINT-8 was first introduced in 2019 and consists of 4 domains (physical, social, mental, positive) and 8 details (stair climbing, pain, energy, working, depression, memory, sleep, happiness). Since there are 4 levels of questions in each domain, the number of health status that could be expressed is richer than that of EQ-5D-5L. In addition, HINT-8 has a lower ceiling effect compared to EQ-5D [18], so it can show the general public’s health-related quality of life more precisely. Because the scores provided for readability were reverse-coded in this study, a higher score indicates a higher health-related quality of life. The total HINT-8 is the result of summing the reverse-coded values of 8 items, with a higher score indicating a higher health-related quality of life [19].

#### 2.2.4. OSA Risk Factors

In 2019, OSA was newly introduced for those over the age of 40. The three risk factors for OSA, snoring, fatigue, and having an eyewitness to sleep apnea, were all rated as ‘yes’ and ‘no’. Those without all the three factors were classified as CG (control group), and those with at least one of the three factors were classified as OSARG (obstructive sleep apnea risk factors group). In this study, 605 patients were classified under OSARG and 836 patients under CG.

### 2.3. Methodology 

IBM SPSS ver. 21.0 (IBM Co., Armonk, NY, USA) was used to analyze the data, and complex sampling analysis with stratified variables, cluster variables, and weighted values was carried out. 

**Figure 2:**
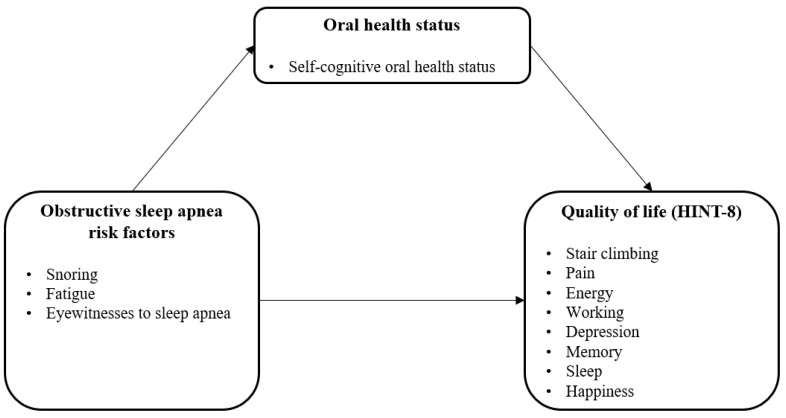
Research model.

A complex sample chi-square test was performed to compare the sociodemographic characteristics, general health condition, and quality of life for 605 patients of OSARG and 836 patients of CG out of the total 1,441 patients. Hierarchical regression was performed to verify the significance of the indirect effect of the independent variable on the dependent variable through the parameter according to the three-step procedure suggested by Baron and Kenny [20]. This measure sought to confirm the mediating effect of self-cognitive oral health status on the impact of OSA risk factors on quality of life. Step 1 is the effect of the independent variable (OSA risk factor) on the parameter (self-cognitive oral health status), step 2 is the effect of the independent variable (OSA risk factor) on the dependent variable (HINT-8), and step 3 is the effect of independent variables (OSA risk factors) and parameters (self-cognitive oral health status) on the dependent variable (HINT-8). Hierarchical regression analysis was performed for each step. In order to increase the reliability of the results, variables reflecting sociodemographic characteristics and general health status that have a significant influence were corrected. Based on the multicollinearity results, the variance inflation factor value was less than 10, indicating that there was no problem (Figure 2). In addition, ‘don’t know’, ‘not applicable’, and ‘missing values’ of 8, 9, 88, and 99 were all excluded from the data, and the number of subjects in all tables was presented with unweighted frequencies. The statistical significance was set at 0.05.

## 3. Results

### 3.1. Sociodemographic Characteristics

Results of confirming the sociodemographic characteristics according to the presence or absence of OSA showed that both groups were often in their 50s, married, and in terms of education, have high school graduation as the highest level of education. CG had the highest income level of ‘high’, while OSARG had the highest income level of ‘middle’. In the case of economic activity, both groups had ‘employment status’ the most. In terms of alcohol consumption, the majority of both groups did not drink at all, while non-smoking status was the most frequent. There were significant differences only in education level and smoking status between the two groups (*p* < 0.05) (Table 1).

### 3.2. Systemic Health Status and Self-Cognitive Oral Health Status According to the Two Groups

Results of checking the systemic health status according to the presence or absence of OSA risk factors showed that 0.3% of the people in the OSARG were diagnosed with OSA. Hypertension, dyslipidemia, diabetes, cervical cancer, depression, allergic coryza, sinusitis, and tympanitis were all higher in OSARG than in CG. Body mass index and neck circumference were also higher in OSARG than in CG, but CG was higher than OSARG for subjects at menopausal age and in relation to self-cognitive oral health status. There were significant differences between the two groups only in terms of dyslipidemia, depression, body mass index, neck circumference, and self-cognitive oral health status (*p* < 0.05) (Table 2).

### 3.3. HINT-8 According to the Two Groups

Results of checking HINT-8 according to OSA risk factors showed that CG was higher than OSARG in all items: stair climbing, pain, energy, working, depression, memory, sleeping, and happiness. The highest item in CG was ‘working’, while ‘happiness’ was the lowest. Meanwhile, the highest items in OSARG were ‘stair climbing’ and ‘working’, while the lowest was ‘happiness’ (*p* < 0.001) (Table 3).

### 3.4. Mediating Effect of Self-Cognitive Oral Health Status on the Effect of OSA Risk Factors on HINT-8

Results of examining the mediating effect of self-cognitive oral health status on the influence of OSA risk factors on HINT-8 showed that OSA risk factors and self-cognitive oral health status were significant (β = −0.072 *p* = 0.018) in stage 1, while OSA risk factors were significant with HINT-8 (β = − 1.329, *p* < 0.001.), meeting the second condition. In the last stage, OSA risk factors were significant with HINT-8 (β = −1.280 *p* < 0.001), while self-cognitive oral health status, which is a parameter, was significant (β = 0.713, *p* < .001). In terms of the influence of OSA risk factors on HINT-8 in stage 2, the β coefficient value was −1.329, whereas the β coefficient value in stage 3 including self-cognitive oral health status was reduced to −1.280, indicating that it was partially mediated (*p* < 0.05) (Table 4).

## 4. Discussion

Middle-aged women make up 31.4% of the Korean population, and this proportion is continuously increasing every year [21]. Middle-aged women’s quality of life related to health is reported to be relatively low compared to other age groups, so it is necessary to pay attention to their quality of life [22]. Although menopause in middle-aged women is a natural change, the quality of life decreases as daily life becomes difficult due to the symptoms and diseases that appear during this period [23]. In particular, the quality of sleep decreases as the OSA risk factors increase due to physical symptoms and emotional changes. The most common symptom and sign that can make OSA clinically suspect is snoring. In addition, apnea can be reported by cohabitants, and OSA patients may complain of fatigue due to frequent awakening and frequent night urination [24,25,26]. Therefore, if there are witnesses to confirm snoring, fatigue, and apnea during sleep, it would be important to recognize the symptoms as risk factors for OSA and detect the condition early. Until now, there have been few studies on risk factors, most of the subjects are not OSA patients, and studies on middle-aged women are insufficient. Therefore, this study analyzed the OSA risk factors, which comprise a comprehensive concept. In this study, only 0.3% of middle-aged women were diagnosed with OSA despite having a risk factor for OSA. This is significantly lower than Australia’s OSA incidence rate of 10–42% [27]. In the case of middle-aged women in Korea, this result is attributed to the inability to recognize it as a disease or the avoidance of embarrassment despite having an OSA risk factor. Therefore, if OSA is expanded as a risk factor rather than analyzing only the diagnostic results of OSA, it can help prevent OSA from progressing.

Results of this study showed that smoking is highly related to OSA risk factors, which may can be considered to prevent OSA from becoming severe. According to a study by Kim et al. [28], tobacco components excessively secrete calcitonin gene-related proteins, causing edema in the mucous membrane. It is reported that the passageway leading to the larynx through the roof of the mouth, oral cavity, and nasal passages stretch more than normal, consequently obstructing airflow, causing more severe snoring and subsequently more severe OSA. Therefore, for smokers among middle-aged women with OSA risk factors, behavioral control by not smoking would be very important.

OSA is dangerous because it is accompanied by various systemic diseases. Irregular breathing and frequent arousal in OSA patients are known to alter physiological activity, causing cardiovascular and metabolic diseases [29,30]. In addition, there are reports that it acts as a risk factor for developing new diseases [29,30]. In this study, dyslipidemia and depression were identified as general health conditions related to OSA risk factors, which is different from previous studies. This difference can be attributed to the fact that in this study, subjects were limited to middle-aged women and not only OSA patients but people with OSA risk factors. Accordingly, since there is a possibility that people with OSA risk factors may present dyslipidemia and depression, it is necessary to prevent progression to other systemic diseases through in-depth examinations and checkups.

In many studies [31,32], OSA also tends to increase when neck circumference, waist circumference, or BMI increase. The results of this study also showed that BMI and neck circumference were highly related to OSA. This is presumed to be due to an increase in fat in the upper respiratory tract, the place where breathing difficulties occur, and narrowing of the airway increasing stenosis [31]. However, there are also studies [33,34] stating that there is no clear association between OSA and neck circumference, so it is necessary to perform an in-depth analysis by identifying many variables in middle-aged women. Nevertheless, the guidelines of the American Academy of Sleep Medicine (AASM) recommend weight loss through diet as a treatment for obese OSA patients [32]. Therefore, in the case of obese middle-aged women who have OSA risk factors, diet and weight control would prevent OSA from progressing to a moderate level. In the same vein, middle-aged women have an increased risk of OSA due to weight gain after menopause. The age of menopause was lower in the group with risk factors for OSA compared to CG, but there was no significant difference. This may be because potential OSA patients were included since risk factors were used, not patients diagnosed with OSA. Therefore, it will be necessary to further investigate only women who have undergone menopause in the future.

As mentioned above, OSA is highly correlated with oral health status [14,15]. Based on the results of checking self-cognitive oral health status in this study, those with OSA risk factors recognized that their oral health status was worse. The self-cognitive oral health status in this study is based on a subject’s perspective. In the study of Cha and Jang [35], it was found that subjective oral health status reflects objective oral health status. Accordingly, in the case of people with OSA risk factors, it would be necessary to closely investigate the oral conditions and oral health behaviors that may affect OSA. In addition, Lim [36] reported that the oral health indicators evaluated by the individual had a greater effect on the quality of life than the oral health indicators evaluated by experts. In previous studies, self-cognitive oral health status was found to be closely related to quality of life [36,37]. In addition, OSA was reported to be highly correlated with quality of life [4,5]. Therefore, this study confirmed the mediating effect of self-cognitive oral health status on the effects of OSA risk factors on quality of life. The partial mediating effect of self-cognitive oral health status was confirmed after correcting sociodemographic characteristics and systemic diseases. Therefore, if there are OSA risk factors among middle-aged women, it would be helpful to manage not only OSA treatment but also oral health to improve quality of life. 

The limitations of this study are as follows. It is difficult to confirm a causal relationship because it is a cross-sectional study, not a cohort study, and there may be research bias because many covariates such as nutritional surveys could not be controlled. In addition, objective oral health data were not used. Therefore, future analysis using objective oral data (oral structure and oral health behavior) that are important for OSA would be needed. Nevertheless, this study is considered to be meaningful as it is the first study to confirm the partial effect of self-cognitive oral health status on OSA risk factors and their influence on quality of life using data from the Korea National Health and Nutrition Examination Survey. In particular, middle-aged women often overlook this issue despite having a risk factor for OSA, so in order to improve quality of life, they should try to improve their oral health condition and take measures to promote early detection and treatment of OSA.

## 5. Conclusions

In this study, it was confirmed that the OSA risk factors for middle-aged women in Korea have a partial effect on quality of life. Therefore, for middle-aged women with OSA risk factors, it will be helpful to improve their quality of life if, in addition to OSA disease treatment, oral health management is performed. In middle-aged women, there is a lack of awareness of OSA risk factors or a tendency to avoid treatment, making early detection of OSA difficult. It is necessary to continuously inform the public of the risk of OSA and conduct regular oral checkups to improve quality of life.

## Figures and Tables

**Table 1:** Demographic characteristics according to the two groups. Unit: N (%).

Characteristics	Division	CG (*N* = 836)	OSARG (*N* = 605)	*p* *
Age	40–49	331 (41.5)	223 (39.9)	0.564
	50–59	345 (42.4)	245 (41.8)	
	60–64	160 (16.1)	137 (18.3)	
Marriage status	Single	32 (3.1)	20 (3.3)	0.861
	Married	804 (96.9)	585 (96.7)	
Education level	≤Elementary school	71 (7.5)	74 (11.2)	0.013
	Middle school	75 (8.2)	72 (10.1)	
	High school	347 (42.5)	253 (43.4)	
	≥College	343 (41.9)	205 (35.3)	
Income level	Low	139 (15.7)	130 (19.8)	0.127
	Low-middle	165 (20.3)	114 (19.5)	
	Middle	161 (18.7)	133 (22.3)	
	Middle-high	183 (22.4)	110 (18.8)	
	High	186 (22.9)	114 (19.5)	
Economic activity	Employed	516 (62.4)	353 (57.4)	0.078
	Unemployed	320 (37.6)	252 (42.6)	
Drinking	Not at all	309 (53.9)	215 (52.2)	0.931
	Less than once a month	119 (20.5)	82 (20.6)	
	About once a month	76 (14.2)	62 (14.1)	
	Once a week	51 (9.0)	38 (10.9)	
	Almost everyday	17 (2.5)	10 (2.3)	
Smoking	Non-smoking	806 (96.1)	563 (92.4)	0.019
	Smoking	27 (3.9)	41 (7.6)	

* using complex sample chi-square test, *p* < 0.05.

**Table 2:** Systemic health status according to the two groups. Unit: N (%).

Characteristics	CG (N = 1295)	OSARG (N = 1127)	*p* *
* OSA (have, %)	0 (0.0)	2 (0.3)	0.113
* High blood pressure (have, %)	138 (16.2)	119 (19.9)	0.092
* Dyslipidemia (have, %)	156 (18.4)	136 (23.4)	0.033
* Diabetes (have, %)	41 (4.9)	44 (6.6)	0.191
* Cervical cancer (have, %)	4 (0.4)	4 (0.8)	0.305
* Depression (have, %)	41 (4.9)	59 (9.1)	0.005
* Allergic rhinitis (have, %)	137 (15.9)	117 (20.5)	0.066
* Sinusitis (have, %)	42 (4.5)	41 (6.5)	0.153
* Otitis media (have, %)	41 (4.5)	30 (5.4)	0.451
^†^Body mass index	23.28 ± 0.15	23.84 ± 0.18	0.009
^†^Neck circumference	32.59 ± 0.10	32.99 ± 0.10	<0.001
^†^Menopause age	50.11 ± 0.20	49.82 ± 0.27	0.385
^†^Self-awareness of oral health status	2.78 ± 0.03	2.64 ± 0.03	0.001

* using complex sample chi-square test, ^†^using complex sample linear regression analysis, *p* < 0.05.

**Table 3:** HINT-8 according to the two groups.

Characteristics	CG (*N* = 1295)	OSARG (*N* = 1127)	*p* *
1. Climbing stairs	3.63 ± 0.02	3.37 ± 0.03	<0.001
2. Pain	3.44 ± 0.02	3.19 ± 0.03	<0.001
3. Energy	3.12 ± 0.03	2.65 ± 0.40	<0.001
4. To work	3.67 ± 0.20	3.37 ± 0.03	<0.001
5. Depressed	3.60 ± 0.02	3.31 ± 0.03	<0.001
6. Memory	3.52 ± 0.02	3.34 ± 0.02	<0.001
7. Sleep	3.52 ± 0.02	3.25 ± 0.03	<0.001
8. Happiness	2.88 ± 0.03	2.59 ± 0.04	<0.001
HINT-8	27.37 ± 0.12	25.07 ± 0.14	<0.001

* using complex sample linear regression analysis. *p* < 0.05.

**Table 4:** Mediating effect of self-cognitive oral health status on the effects of OSA risk factors on HINT-8.

Step	Model	β	t	*p* *
1	OSA risk factors → self-awareness of oral health status	−0.072	−2.401	0.018
2	OSA risk factors → HINT-8	−1.329	−9.268	<0.001
3	OSA risk factors → HINT-8	−1.280	−8.690	<0.001
	Self-awareness of oral health status → HINT-8	0.713	5.944	

* using hierarchical regression analysis. *p* < 0.05, 1s R2 = 0.027, 2s R2 = 0.175, 3s R2 = 0.200.

## Data Availability

Not applicable.

## Abbreviations

OSA; Obstructive Sleep Apnea; KNHANES; Korea National Health and Nutrition Examination Survey; OSARG; Obstructive Sleep Apnea Risk Factors Group.
